# Reactive Oxygen and Nitrogen Species Regulate Key Metabolic, Anabolic, and Catabolic Pathways in Skeletal Muscle

**DOI:** 10.3390/antiox7070085

**Published:** 2018-07-05

**Authors:** Roland Nemes, Erika Koltai, Albert W. Taylor, Katsuhiko Suzuki, Ferenc Gyori, Zsolt Radak

**Affiliations:** 1 Faculty of Sports and Health Studies, Hosei University, Tokyo 194-0298, Japan; nem_roland@hosei.ac.jp; 2 Research Institute of Sport Science, University of Physical Education, Alkotas u. 44, H-1123 Budapest, Hungary; koltaie@gmail.com; 3 Faculty of Health Sciences, The University of Western Ontario, London, ON N6G 1H1, Canada; ataylor2@uwo.ca; 4 Faculty of Sport Sciences, Waseda University, Saitama 359-1192, Japan; katsu.suzu@waseda.jp; 5 Institute of Sport Science, University of Szeged, H-6726 Szeged, Hungary; gyori@jgypk.szte.hu

**Keywords:** training, skeletal muscle adaptation, oxidative stress

## Abstract

Reactive oxygen and nitrogen species (RONS) are important cellular regulators of key physiological processes in skeletal muscle. In this review, we explain how RONS regulate muscle contraction and signaling, and why they are important for membrane remodeling, protein turnover, gene expression, and epigenetic adaptation. We discuss how RONS regulate carbohydrate uptake and metabolism of skeletal muscle, and how they indirectly regulate fat metabolism through silent mating type information regulation 2 homolog 3 (SIRT3). RONS are causative/associative signaling molecules, which cause sarcopenia or muscle hypertrophy. Regular exercise influences redox biology, metabolism, and anabolic/catabolic pathways in skeletal muscle in an intensity dependent manner.

## 1. Introduction

Regular physical exercise results in systemic adaptation(s) of the whole body, alongside varied perturbations in blood flow and metabolism among different organs. The main adaptive effects, such as decreased levels of oxidative damage, increased activities of enzymatic antioxidants, enhanced mitochondrial efficiency, and more efficient physiological functions are, however, observed in the skeletal muscle, heart, brain, liver, kidneys, and testes, alongside other organs. Since skeletal muscle is the largest organ in the human body, and the main organ responsible for physical exercise, the present review focuses on exercise-associated adaptation of skeletal muscle, and reactive oxygen and nitrogen species (RONS).

## 2. Muscle Contraction and Reactive Oxygen and Nitrogen Species

Davies and co-workers [1] showed for the first time that after an exhaustive bout of exercise, a significant increase in reactive oxygen species (ROS) production occurs in the skeletal muscle, as measured by electron spin resonance. At that time, it was widely accepted that ROS were a so called “by-product” of aerobic metabolism that jeopardized the structure and function of muscle cells. However, an intriguing study was later published in which it was observed that whilst contracting skeletal muscle generates ROS, ex vivo exposure to the antioxidant enzymes, catalase and superoxide dismutase (SOD), decreased force generation [2]. This work was the first to show that ROS could positively affect the function of skeletal muscle by facilitating muscle contraction at certain concentrations. In a previous study, the same research group showed that during muscle fatigue the concentration of ROS increased, which eventually led to a decreased force production, which could be delayed with the administration of exogenous antioxidants [2,3,4]. Therefore, this finding suggests that contracting skeletal muscle is producing ROS, which further facilitates the strength of the muscle contraction. However, if the ROS concentration exceeds a certain level, it reduces the force generation and causes fatigue. This phenomenon nicely demonstrates that ROS could have a positive or negative effect, depending on the concentration, and is a phenomenon that can be described by the hormesis curve [5,6,7,8]. Up-to a concentration, ROS and muscle force generation capacity increase together, but after reaching a point, greater levels of ROS decrease the force generation of skeletal muscle, and, therefore, the relationship between ROS and force generation of skeletal muscle has a bell-shaped dose-response curve.

**Figure 1:**
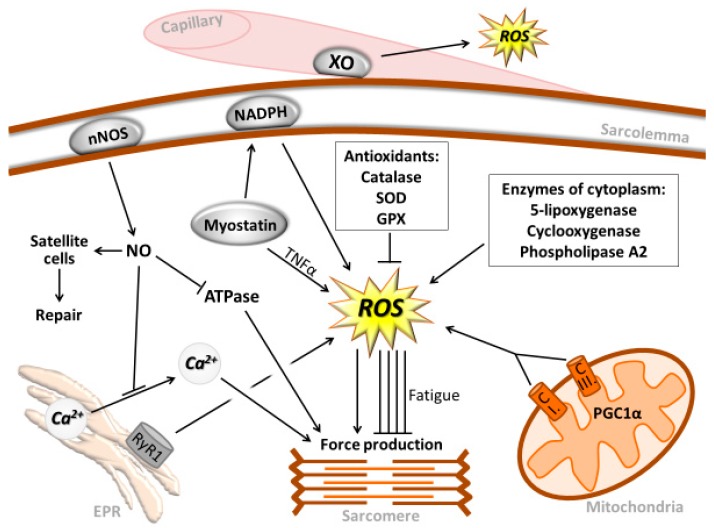
Sources of reactive oxygen species. Summarized potential sources of reactive oxygen species (ROS) in skeletal muscle. H_2_O_2_ up to a certain concentration enhance the force production of skeletal muscle, while in large concentrations, ROS causes fatigue and suppresses force generation.

It is known that mitochondrial electron transport chain is one main ROS generator found in skeletal muscle [9]. During high intensity exercise, ROS, mainly generated by Complex I and III with pyruvate/malate and succinate substrates, were increased by 187% and 138%, respectively [10]. Experimental data revealed that mitochondria isolated from skeletal muscle after contraction showed significantly increased levels of hydrogen peroxide (H_2_O_2_) generation [11] (Figure 1). H_2_O_2_ is the main signaling molecule because it can cross membranes and activate redox sensitive proteins, modulating cell signaling. 

It has been shown that Complex I is the major ROS generator in skeletal muscle of ultra-endurance runners [12]. However, it must be noted that the earlier estimations of about 1–5% of the oxygen that entering the mitochondria being released as ROS [13] could be highly overestimated, with the real value being more than an order of magnitude lower [14]. However, the findings of Austin et al. [15] suggest that mitochondria might be important sources of ROS at Complex I and III, through peroxisome proliferator-activated receptor-γ coactivator alpha (PGC-1α). 

Indeed, the iron-sulfur clusters, flavoprotein and oxidoreductase, at Complex I, and Q10 semiquinones at Complex III are thought to be the main sites of ROS generation [14,16]. In addition to mitochondria, 5-lipoxygenase, cyclooxygenase, sarcolemmal and leukocyte nicotinamide adenine dinucleotide phosphate-oxidase (NADPH), and xanthine oxidase (XO) have also been implicated in superoxide generation in skeletal muscle [9,17,18,19].

It has also been suggested that the basal level of intracellular H_2_O_2_ in skeletal muscle is between 10–100 nM, which increased to 100–200 nM with heavy muscle contraction [20]. Although the level of XO in skeletal muscle is very low, it is present to a significant degree in the endothelium, and, hence, it is a potential source of extracellular superoxide generation. High intensity exercise results in the generation of hypoxanthine [21,22], and a linear relationship has been observed between the levels of circulating lactic acid and XO. Interestingly, we could detect increased XO activity in the liver one day after exhaustive acute exercise [23], but administration of SOD derivatives identified endothelium associated with XO as one source of ROS generation during intense exercise [21]. However, the contribution of XO in ROS production during aerobic exercise is a real puzzle. A number of papers suggest that allopurinol administration can attenuate ROS production during aerobic exercise, and, moreover, allopurinol can even prevent the ROS associated adaptive responses to exercise [24].

Contraction of skeletal muscle results in ROS generation associated with phospholipase A (PLA_2_). Indeed, ROS production decreased when various PLA_2_ inhibitors were administered to a contracting diaphragm, suggesting that PLA_2_ plays a critical role in modulating ROS formation during muscle contraction [25] (Figure 1). Furthermore, it has been suggested that, in the skeletal muscles of patients suffering from Duchenne muscular dystrophy, elevated intracellular calcium levels caused by altered regulation of calcium channels activate PLA_2_, causing ROS production and increased membrane permeability [26]. In Duchenne muscular dystrophy, dystrophin is absent, and sarcolemmal neuronal nitric oxide synthase (nNOS) is lost because it is anchored to dystrophin. The absence of nNOS-generated NO could be one of the reasons for increased ROS generation by skeletal muscle of patients with this atrophy. Indeed, when nNOS is knocked out, higher levels of intrinsic hydrogen peroxidase activity were demonstrated in the extensor digitorum longus (EDL) of nNOS-knockout mice, when compared to C57 control mice [27]. Therefore, nNOS-generated NO could have an important scavenging role in the detoxification of superoxide. Interestingly, Ca^2+^-independent (iPLA_2_) beta is involved in membrane repair; suggesting that the interaction between ROS and membrane lipids not only disrupts the cellular milieu and jeopardizes the fate of the cell, but could also be important for the continuous remodeling requirements of cell membranes [28].

Recently, myostatin emerged as a potential ROS-inducing factor, especially during sarcopenia [29]. It has been demonstrated that knocking out the myostatin gene resulted in attenuated loss of muscle mass with aging, and that myostatin can induce ROS production through tumor necrosis factor-α (TNF-α) and NADPH oxidase [29]. However, the role of exercise in myostatin-mediated redox signaling is still unclear and further research is warranted on this topic. Activation of ryanodine receptor 1 (RyR1) in the sarcoplasmic reticulum of skeletal muscle is necessary for Ca^2+^ release and the subsequent generation of cross-bridge-related force production. With the aging of skeletal muscle, a continuous Ca^2+^ leak is observed in RyR1 channels, which is associated with a decreased force production capacity and increased ROS production. Pharmacological intervention to normalize RyR1 function by stabilizing the binding of calstabin1 to RyR1 significantly reduced Ca^2+^ leakage and increased endurance capacity [30].

It has been shown that single and regular bouts of exercise differentially modulate ROS production in neutrophils. During acute exercise the adaptive response is limited, and, indeed, it has been shown that a single bout of exercise results in a loss of mitochondrial membrane potential, e [31]. Marathon running, which is a severe form of exercise, caused cytokines and neutrophil activation markers (myeloperoxidase (MPO) and lactoferrin (LTF), and priming neutrophils and monocytes were secreted and functional after exhaustive exercise [32]. These responses seemed to be overwhelming, inducing antioxidant and anti-inflammatory defenses systems, and preventing exercise-induced oxidative stress [32]. Therefore, it is quite clear that a single bout of exhaustive exercise induces inflammation [33,34] which can readily lead to oxidative stress.

Muscle contraction generates heat, which has been shown to enhance ROS production [35]. However, ROS production is an essential physiological process for muscle contraction, where it is estimated that H_2_O_2_ concentrations can increase by 100 nM during contractions [20]. Indeed, whilst it is known that low levels of exogenous H_2_O_2_ treatment increase force production, e.g., in the diaphragm, the addition of catalase decreases diaphragm force production [2]. This response has been associated with H_2_O_2_ modulating muscle contraction via Ca^++^ channels [36].

Moreover, it appears that not only Ca^2+^ sensitivity, but also the release of Ca^2+^ is altered by oxidants [37]. On the other hand, it is also known that Ca^2+^-ATP-ase activity of the sarcoplasmic reticulum is easily depressed by H_2_O_2_ [38]. Whilst not completely clear, current information suggests that the physiological regulatory role of H_2_O_2_ is more significant than that of superoxide or hydroxyl radical due to the very shot half-life of the latter two [39]. Furthermore, H_2_O_2_ can cross the cell membrane, while superoxide (O•^−^_2_) and hydroxyl radicals (•OH) apparently cannot.

NO could also affect the function of skeletal muscle. The synthesis of NO is catalyzed by the enzyme, NOS. NOS converts arginine and molecular oxygen to NO and citrulline in a reaction that requires NADPH, flavin adenine dinucleotide, flavin mononucleotide, and tetrahydrobiopterin as cofactors. The predominant NOS isoform in skeletal muscle is neuronal NOS (nNOS), although skeletal muscle also expresses endothelial NOS (eNOS) and inducible NOS (iNOS). The nNOS is present in the sarcolemma of both extra- and intra-fusal muscle fibers. In addition, nNOS is concentrated at the postsynaptic surface of the mammalian neuromuscular junction of all fibers. Also, whilst eNOS is abundant in skeletal muscle vasculature, iNOS is present at low levels in rodent and human skeletal muscles, and is localized to the sarcolemma through caveolin-3. NO could influence neuromuscular transmission and act as a retrograde signal to modify pre-synaptic function [40]. The effect of NO on contractile function is better known and studied than its role in neuromuscular transmission. However, observations have revealed that NO decreases isometric force, and, in general, decreases force production in skeletal muscle [41]. One of the reasons behind this phenomenon could be that actomyosin ATP-ase activity is reduced by nitrosylation, which is mediated by NO, and results in a decreased force production [42]. Moreover, NO can inhibit Ca^2+^ release from the sarcoplasmic reticulum [43], resulting in a decreased force production. Whilst muscle soreness is associated with marked decreases in maximal force generation, we have shown that NO could be one of the factors that is responsible for this. Significant increases have been shown in NO content with muscle soreness, which correlates with a decreased maximal force production [44]. We hypothesized that an increased NO level with muscle soreness could be a protective mechanism that does not allow high force production, which, due to high muscular tension, can lead to the development of micro-injuries. In addition, elevated levels of NO during muscle contraction could also be responsible for pain. This is a result of NO activating nociceptors that host the calcitonin gene-related peptide (CGRP) receptor, which is activated by NO, thus, causing pain. Moreover, it is well known that muscle soreness causes damage to sarcomeres due to the unaccustomed tension. The damage must be repaired and it appears that NO is involved in the repair process by activating satellite cells [44]. NO induces satellite cell proliferation, which is a crucial process in muscle repair; a process which is absent in patients with Duchenne muscular dystrophy due to a lack of nNOS.

NO is able to significantly interfere with cellular metabolism by decreasing oxygen consumption [45], altering glucose uptake, and controlling vasodilation. With muscle injury and inflammation, NO is generated to a greater extent by macrophages, mostly through the iNOS process. Nuclear factor kappa B (NF-kB), which is one of the master regulators of inflammation by the regulation of transcription of a number of inflammatory proteins, could also regulate the expression of iNOS. NF-kB is a redox sensitive transcription factor, which, besides its role in inflammation, could also alter the transcription of manganese-SOD (MnSOD) [46].

## 3. RONS-Associated Oxidative Damage and Repair

RONS are very potent inducers of the enzymatic antioxidant system. The extent of oxidative damage reveals the efficiency of antioxidant and oxidative damage repair systems, although it is intriguing that the level of oxidative damage is never zero. This could indicate that the damage might have some physiological roles, such as signaling [46]. It is known that during DNA replication generated errors initiate the repair process, and similar phenomena could happen with the oxidant-generated damage to DNA. The study of skeletal muscle is limited with regards to the effects of RONS. Nonetheless, adequate literature is available to suggest that more studies should be carried out, especially within exercise models. For example, the activity of 8-oxoguanine-DNA glycosylase 1 (OGG1) increased in human rectus femoris muscle after a marathon race [47]. We suggested that the level of 8-oxodeoxyguanosine (8-oxodG) increased in the muscle because of the exercise, which was followed by the induction of the enzyme necessary to repair the mutagenic damage. We have also reported that aging results in an increased level of nuclear 8-oxodG in the skeletal muscle of rats. This increase was prevented by exercise training and the induction of OGG1 [48]. This result led to an interest in measuring the activity of OGG1 and uracil DNA glycosylase (UDG) in white and red portions of the quadriceps muscle [49] because Type I and Type II fibers differ greatly in metabolic rate, as well as in their levels of antioxidant capacity. We found that OGG1 activity increased in the nuclei of red fibers as was expected; but, surprisingly, OGG1 activity decreased in the mitochondria of both red and white fibers. We were puzzled by this phenomenon and, in a related study, found that the export of OGG1 to the mitochondria could be accelerated by exercise training [50]. In other words, a sedentary life-style and/or detraining impairs the transfer of OGG1 into the mitochondrial matrix [50]. We suggest that exercise training results in biogenesis of mitochondria, and provides more accessible membranes for proteins to be transferred into mitochondria after their synthesis in the ribosomes.

It is well demonstrated that red fibers with high oxidative capacity host a substantial enzymatic antioxidant system, and express increased resistance to oxidative stress; whereas white fibers do not. The activity of OGG1 is also higher in red fibers, but significant differences in the activity of UDG in different fiber types are not evident. DNA repair enzymes work as house-keeping enzymes and are designed to decrease the level of oxidative damage for the protection of cells, and to avoid apoptosis and necrosis, as well as mutation. Although DNA suffers a significant attack from ROS, the extent of protein damage is one-fold higher [51]. Oxidized proteins are not repaired in the same fashion as DNA, but, to prevent the aggregation and cross-folding of oxidized defective proteins, the proteasome system is the first line of defense. Again, skeletal muscle is not a very well monitored tissue in the case of the proteasome system. It has been suggested that aging, which results in a very significant loss of muscle mass, does not alter the activity of the proteasome system [52,53] or decrease its activity [51,54]. However, there are reports that caloric restriction and exercise training increase the activity of the proteasome system [48,55]. This suggestion could be important for remodeling the tissues and removing damaged proteins. The response of the proteasome system to exercise is dependent on the exercise loading and the time of sampling [56]. Therefore, the findings must be evaluated accordingly. For example, Sultan and his co-workers [57] have shown that chronic low-frequency electrical stimulation, which induces fast-to-slow transitions of muscle fibers, alters the proteasome system, once again demonstrating the plasticity of skeletal muscle. Lipid peroxidation is not repaired as efficiently as DNA damage (that damage repaired first, which most directly affects the fate of the cell), but iPLA_2_ beta can repair lipid damage to a certain degree. We have suggested that the limited extent of DNA damage is an important stimulator of gene expression, protein damage, protein turn-over, and lipid damage, and could be important for membrane remodeling [28].

## 4. The Role of ROS in Exercise-Induced Metabolism

**Figure 2:**
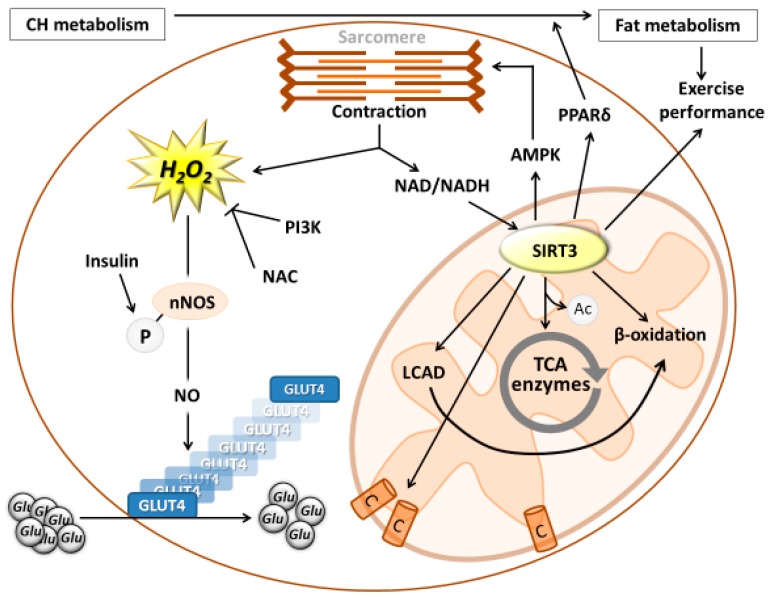
The role of reactive oxygen species on metabolism. H_2_O_2_ can stimulate cellular signaling pathways to dislocate GLUT4 to cellular membranes, which is crucial for glucose uptake. ROS levels influence the activity of SIRT3, which is an important regulator of fat metabolism.

During muscle contraction, there is a significant change in intracellular redox levels, and H_2_O_2_ concentration is elevated to 100–200 nM. However, an intriguing question is whether this could help cover the energy cost of muscle contraction. An early study on L6 myotubes showed that H_2_O_2_ increased the mRNA levels of glucose transporter 1 (GLUT1) and the glucose uptake of these cells [58]. Further, a later study on mouse skeletal muscle showed that repeated contractions increased 2-deoxyglucose (2-DG) uptake roughly threefold in isolated EDL (fast-twitch) muscle. N-Acetylcysteine (NAC), a non-specific antioxidant, inhibited contraction-mediated 2-DG uptake by approximately 50% (*p* < 0.05) compared with control values), yet did not significantly affect basal 2-DG uptake [59]. This suggests that elevated levels of H_2_O_2_ stimulate glucose uptake of skeletal muscle during exercise [59]. A follow-up study on isolated rat EDL muscle revealed that H_2_O_2_ also resulted in a dose-dependent increase in 2-DG uptake in isolated EDL muscles, and the maximal increase was threefold over basal levels at a concentration of 600 μM/L H_2_O_2_. H_2_O_2_-stimulated 2-DG uptake was completely inhibited by the phosphatidylinositol 3-kinase (PI3K) inhibitor, wortmannin, indicating a crucial role of the PI3K pathway in H_2_O_2_-mediated glucose uptake during contractions [60]. In addition, it has been reported that H_2_O_2_ induces phosphorylation of nNOS at the same residue as insulin does, but also stimulates NO production and GLUT4 translocation. nNOS inhibition prevented H_2_O_2_-induced GLUT4 translocation [61] (Figure 2). Moreover, inhibition of AMP activated protein kinase (AMPK) prevented H_2_O_2_ activation and phosphorylation of nNOS, leading to a reduction in NO production and significantly attenuated GLUT4 translocation [61]. It is important to note that acute exposure to H_2_O_2_ or NO increases insulin sensitivity and glucose uptake, while chronic exposure suppresses it, and can easily lead to type 2 diabetes [62]. A significant feature of physical exercise is the cyclic change of adaptive resting periods and exercise periods, with enhanced metabolic processes and ROS production. This cyclic nature is an important part of preconditioning. With increasing exercise intensity, there is an increased dependence on carbohydrate metabolism, which parallels the increased generation of ROS. This is unlikely to be a coincidental phenomenon, based on the direct relationship between H_2_O_2_, NO levels and glucose uptake of skeletal muscle.

On the other hand, a pertinent question regarding fat metabolism is whether ROS also regulate the availability of free fatty acids (FFA), and the metabolism of FFA in the TCA cycle. Data suggest that ROS can indirectly regulate the efficiency of fat metabolism through the enzyme silent mating type information regulation 2 homolog 3 (SIRT3), which is localized in the mitochondria, and NAD-dependent lysine deacetylase. In resting human muscle, total NAD^+^ and NADH concentrations are estimated to be ~1.5–1.9 and ~0.08–0.20 mmol/kg dry weight of muscle, respectively [63]. Low intensity muscle contraction increases NAD/NADH concentration. However, the increase in the mitochondrial NAD^+^/NADH ratio during the same absolute exercise seems lower in trained rats [64]. The NAD/NADH ratio reflects the redox state of cell or cellular compartments, which directly affects redox sensitive cellular processes, including metabolism, and particularly those enzymes that are dependent on the availability of NAD. SIRT3 is a NAD-dependent mitochondrial enzyme important for ATP production, since it deacetylates and activates a number of key enzymes in the TCA cycle. Indeed, when nicotinamide riboside, a precursor of NAD^+^ biosynthesis, was supplemented, high fat, diet induced, nonalcoholic fatty liver disease was reverted, partly by the induction of hepatic β-oxidation and mitochondrial complex content and activity [65]. Moreover, when SIRT3 is knocked-out, there is a marked reduction of fatty acid metabolism due to the hyperacetylation of long-chain acyl coenzyme A dehydrogenase (LCAD) at lysine 42 [66]. These results suggest that SIRT3 is a potential regulator of fat metabolism. We, and others, have shown that exercise increases the level of SIRT3 in humans [67,68] and animals [69].

It has been demonstrated that transgenic mice with enhanced levels of SIRT3 in skeletal muscle exhibit 45% better running-based exercise performance than control animals [70]. In addition, these transgenic animals display a higher proportion of slow oxidative muscle fibers, increased muscle AMPK activation, and peroxisome proliferator-activated receptor delta (PPARδ) expression; both of which are known regulators promoting type I muscle fiber specification [70]. Up-regulation of PPARδ might be important because it can lead to a shift from glucose metabolism to fat metabolism, since PPARδ activation potently suppresses glucose catabolism, without affecting either muscle fiber type or mitochondrial content [71]. Higher levels of aerobic endurance capacity are associated with greater utilization of fatty acids. Indeed, it has been shown that mice with an overexpression of PPARδ had better endurance performance than control mice [71]. Overall, it is well demonstrated that redox sensitive SIRT3 activates the metabolism of fat, and, therefore, can influence exercise performance. 

## 5. Role of ROS in Muscle Hypertrophy and Atrophy

The role of ROS in sarcopenia has seemingly been known for quite some time [72]. However, that role has recently been questioned. Transgenic mice expressing a proof-reading-deficient version of PolgA, the nucleus-encoded catalytic subunit of mitochondrial DNA polymerase, were generated [73]. These mice carried a three- to five-fold increase in the levels of point mutations, as well as increased amounts of deleted mitochondrial DNA [73]. As the consequence of genetic manipulation, PolG mice showed a premature onset of ageing-related phenotypes, such as weight loss, reduced subcutaneous fat, alopecia, kyphosis, osteoporosis, anaemia, reduced fertility, and heart enlargement [73]. However, PolG mice do not exhibit increased levels of mitochondrial ROS production [74], suggesting that in this transgenic model the premature aging is not a result of enhanced ROS production. On the other hand, when the gastrocnemius muscles of six month and 21 month-old rats were studied, it was shown that mitochondria obtained from aged muscle fibers show several functional abnormalities, explaining the enhancedproteolysis, ROS overproduction, and vulnerability to apoptosis exhibited by sarcopenic muscle [75]. In another transgenic mouse model where the Cu-ZnSOD was ablated, the aging process was accelerated in skeletal muscle, leading to a proposal that superoxide-induced neuromuscular junction degeneration and mitochondrial dysfunction are potential mechanisms of sarcopenia [76].

Systemic age-associated inflammation in the skeletal muscle has also been suggested to be one of the causative factors of loss of muscle strength and mass [77,78,79]. Massive involvement of ROS is suggested in sarcopenia-associated inflammation [78,80], with supporting data coming from a number of studies on aging [81,82,83]. One observed that inflammatory mediator angiopoietin-like protein 2 (ANGPTL2) increases in the skeletal muscle of aging mice, while exercise attenuates this elevation [84]. When we compared markers of anabolic and catabolic processes in the skeletal muscle of five month and twenty-eight month-old rats, we observed that aging resulted in decreased levels of follistatin/mTOR/Akt/Erk activation and increased myostatin/Murf1/2, proteasome subunits, and protein ubiquitination levels. In addition, the TNF-α, ROS, p53, and Bax levels were increased, while Bcl-2 levels were decreased in the skeletal muscle of aged rats [85]. We trained rats at an intensity of 60% of VO2max on a treadmill, and this running training attenuated age-associated increases in apoptotic and catabolic processes [85]. From a muscle hypertrophy perspective, it is difficult to cause muscle hypertrophy in laboratory animals. However, one of the most successful models is wing loading on birds. When young and aged Japanese quails were loaded for seven or 21 days to induce hypertrophy, data showed that H_2_O_2_ content was higher in muscles from aged birds following seven days of loading [86]. Moreover, it appears that loading suppresses pro-apoptotic signaling in quail muscle, but aging delays or attenuates these anti-apoptotic changes [86].

**Figure 3:**
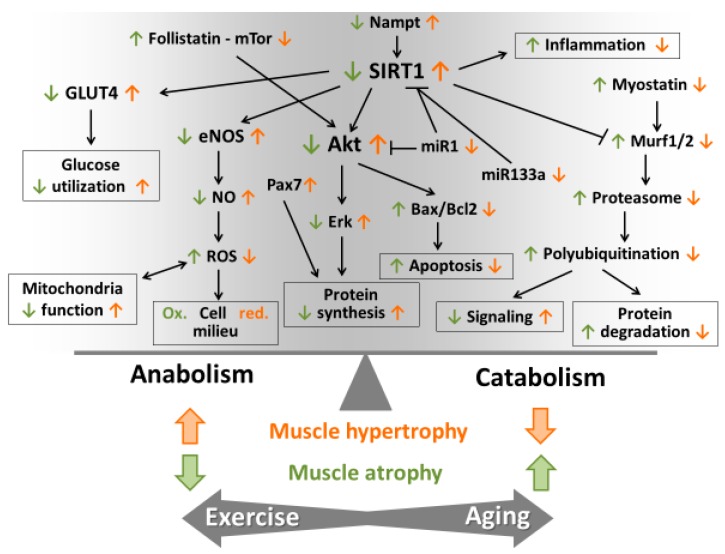
Schematic model of muscle hypertrophy and atrophy. The figure shows the schematic model, the molecular mechanisms of muscle hypertrophy, and age-associated muscle atrophy. SIRT1 is one of the key regulators of anabolic and catabolic processes in skeletal muscle.

We have also studied muscle hypertrophy. Soleus and gastrocnemius muscles were ablated to cause compensatory hypertrophy on plantaris muscle [87]. Two weeks after the removal of soleus and gastrocnemius muscles we observed about a 40% increase in the muscle mass of plantaris muscle. This hypertrophy was associated with a significant increase in silent mating type information regulation 2 homologue 1 (SIRT1) content and activity (*p* < 0.001). SIRT1-regulated Akt, endothelial nitric oxide synthase, and GLUT4 levels. SIRT1 levels were correlated with muscle mass, paired box protein 7 (Pax7), proliferating cell nuclear antigen (PCNA), and nicotinamide phosphoribosyltransferase (Nampt) levels [87] (Figure 3). These data suggest that the redox state of the cells influences muscle growth, at least in this model. We also found that increased levels of K63 and muscle RING finger 2 (MuRF2) protein could also be important enhancers of muscle mass, and reported that the levels of microRNA (miR)1 and miR133a decrease in hypertrophy, and negatively correlate with muscle mass, SIRT1, and Nampt levels. These data suggest a strong correlation between SIRT1 and overload-induced hypertrophy [87].

## 6. Conclusions

Reactive oxygen species are continuously generated in contracting skeletal muscle, and their presence is obligatory for normal physiological function. Besides mitochondrial ROS production, XO and NADPH are the main sources of ROS. Moderate levels of ROS regulate metabolic processes in skeletal muscle, especially carbohydrate metabolism, and are also indirectly involved in fat metabolism through SIRT3. ROS-mediated structural changes of lipids, proteins, and DNA, to a degree, could be important for membrane remodeling, protein turnover, gene expression, or epigenetic regulation. Therefore, ROS are important causative or associative factors for sarcopenia and muscle hypertrophy.

## References

[1] Davies K.J., Quintanilha A.T., Brooks G.A., Packer L. (1982). Free radicals and tissue damage produced by exercise. Biochem. Biophys. Res. Commun..

[2] Reid M.B., Khawli F.A., Moody M.R. (1993). Reactive oxygen in skeletal muscle. III. Contractility of unfatigued muscle. J. Appl. Physiol. (1985).

[3] Reid M.B., Haack K.E., Franchek K.M., Valberg P.A., Kobzik L., West M.S. (1992). Reactive oxygen in skeletal muscle. I. Intracellular oxidant kinetics and fatigue in vitro. J. Appl. Physiol. (1985).

[4] Reid M.B., Shoji T., Moody M.R., Entman M.L. (1992). Reactive oxygen in skeletal muscle. II. Extracellular release of free radicals. J. Appl. Physiol. (1985).

[5] Goto S., Naito H., Kaneko T., Chung H.Y., Radak Z. (2007). Hormetic effects of regular exercise in aging: Correlation with oxidative stress. Appl. Physiol. Nutr. Metab..

[6] Radak Z., Chung H.Y., Goto S. (2005). Exercise and hormesis: Oxidative stress-related adaptation for successful aging. Biogerontology.

[7] Radak Z., Chung H.Y., Goto S. (2008). Systemic adaptation to oxidative challenge induced by regular exercise. Free Radic. Biol. Med..

[8] Radak Z., Chung H.Y., Koltai E., Taylor A.W., Goto S. (2008). Exercise, oxidative stress and hormesis. Ageing Res. Rev..

[9] Powers S.K., Jackson M.J. (2008). Exercise-induced oxidative stress: Cellular mechanisms and impact on muscle force production. Physiol. Rev..

[10] Saborido A., Naudi A., Portero-Otin M., Pamplona R., Megias A. (2011). Stanozolol treatment decreases the mitochondrial ros generation and oxidative stress induced by acute exercise in rat skeletal muscle. J. Appl. Physiol..

[11] Vasilaki A., Mansouri A., Remmen H., van der Meulen J.H., Larkin L., Richardson A.G., McArdle A., Faulkner J.A., Jackson M.J. (2006). Free radical generation by skeletal muscle of adult and old mice: Effect of contractile activity. Aging Cell.

[12] Sahlin K., Shabalina I.G., Mattsson C.M., Bakkman L., Fernstrom M., Rozhdestvenskaya Z., Enqvist J.K., Nedergaard J., Ekblom B., Tonkonogi M. (2010). Ultraendurance exercise increases the production of reactive oxygen species in isolated mitochondria from human skeletal muscle. J. Appl. Physiol..

[13] Boveris A., Chance B. (1973). The mitochondrial generation of hydrogen peroxide. General properties and effect of hyperbaric oxygen. Biochem. J..

[14] St-Pierre J., Buckingham J.A., Roebuck S.J., Brand M.D. (2002). Topology of superoxide production from different sites in the mitochondrial electron transport chain. J. Biol. Chem..

[15] Austin S., Klimcakova E., St-Pierre J. (2011). Impact of pgc-1alpha on the topology and rate of superoxide production by the mitochondrial electron transport chain. Free Radic. Biol. Med..

[16] Muller F.L., Liu Y., Van Remmen H. (2004). Complex III releases superoxide to both sides of the inner mitochondrial membrane. J. Biol. Chem..

[17] Ortenblad N., Young J.F., Oksbjerg N., Nielsen J.H., Lambert I.H. (2003). Reactive oxygen species are important mediators of taurine release from skeletal muscle cells. Am. J. Physiol. Cell Physiol..

[18] Bejma J., Ji L.L. (1999). Aging and acute exercise enhance free radical generation in rat skeletal muscle. J. Appl. Physiol..

[19] Powers S.K., Nelson W.B., Hudson M.B. (2011). Exercise-induced oxidative stress in humans: Cause and consequences. Free Radic. Biol. Med..

[20] Jackson M.J. (2011). Control of reactive oxygen species production in contracting skeletal muscle. Antioxid. Redox. Signal..

[21] Radak Z., Asano K., Inoue M., Kizaki T., Oh-Ishi S., Suzuki K., Taniguchi N., Ohno H. (1995). Superoxide dismutase derivative reduces oxidative damage in skeletal muscle of rats during exhaustive exercise. J. Appl. Physiol..

[22] Xia Y., Zweier J.L. (1995). Substrate control of free radical generation from xanthine oxidase in the postischemic heart. J. Biol. Chem..

[23] Radak Z., Asano K., Inoue M., Kizaki T., Oh-Ishi S., Suzuki K., Taniguchi N., Ohno H. (1996). Superoxide dismutase derivative prevents oxidative damage in liver and kidney of rats induced by exhausting exercise. Eur. J. Appl. Physiol. Occup. Physiol..

[24] Gomez-Cabrera M.C., Domenech E., Vina J. (2008). Moderate exercise is an antioxidant: Upregulation of antioxidant genes by training. Free Radic. Biol. Med..

[25] Nethery D., Stofan D., Callahan L., DiMarco A., Supinski G. (1999). Formation of reactive oxygen species by the contracting diaphragm is PLA_2_ dependent. J. Appl. Physiol (1985).

[26] Allen D.G., Whitehead N.P. (2011). Duchenne muscular dystrophy—What causes the increased membrane permeability in skeletal muscle?. Int. J. Biochem Cell Biol..

[27] Da Silva-Azevedo L., Jahne S., Hoffmann C., Stalder D., Heller M., Pries A.R., Zakrzewicz A., Baum O. (2009). Up-regulation of the peroxiredoxin-6 related metabolism of reactive oxygen species in skeletal muscle of mice lacking neuronal nitric oxide synthase. J. Physiol..

[28] Radak Z., Zhao Z., Goto S., Koltai E. (2011). Age-associated neurodegeneration and oxidative damage to lipids, proteins and DNA. Mol. Aspects Med..

[29] Sriram S., Subramanian S., Sathiakumar D., Venkatesh R., Salerno M.S., McFarlane C.D., Kambadur R., Sharma M. (2011). Modulation of reactive oxygen species in skeletal muscle by myostatin is mediated through nf-kappab. Aging Cell.

[30] Andersson D.C., Betzenhauser M.J., Reiken S., Meli A.C., Umanskaya A., Xie W., Shiomi T., Zalk R., Lacampagne A., Marks A.R. (2011). Ryanodine receptor oxidation causes intracellular calcium leak and muscle weakness in aging. Cell Metab..

[31] Syu G.D., Chen H.I., Jen C.J. (2011). Severe exercise and exercise training exert opposite effects on human neutrophil apoptosis via altering the redox status. PLoS ONE.

[32] Suzuki K., Nakaji S., Yamada M., Liu Q., Kurakake S., Okamura N., Kumae T., Umeda T., Sugawara K. (2003). Impact of a competitive marathon race on systemic cytokine and neutrophil responses. Med. Sci. Sports Exerc..

[33] Suzuki K., Totsuka M., Nakaji S., Yamada M., Kudoh S., Liu Q., Sugawara K., Yamaya K., Sato K. (1999). Endurance exercise causes interaction among stress hormones, cytokines, neutrophil dynamics, and muscle damage. J. Appl. Physiol. (1985).

[34] Kawanishi N., Mizokami T., Niihara H., Yada K., Suzuki K. (2016). Neutrophil depletion attenuates muscle injury after exhaustive exercise. Med. Sci. Sports Exerc..

[35] Zuo L., Christofi F.L., Wright V.P., Liu C.Y., Merola A.J., Berliner L.J., Clanton T.L. (2000). Intra- and extracellular measurement of reactive oxygen species produced during heat stress in diaphragm muscle. Am. J. Physiol. Cell Physiol..

[36] Andrade F.H., Reid M.B., Allen D.G., Westerblad H. (1998). Effect of hydrogen peroxide and dithiothreitol on contractile function of single skeletal muscle fibres from the mouse. J. Physiol..

[37] Favero T.G., Zable A.C., Abramson J.J. (1995). Hydrogen peroxide stimulates the Ca^2+^ release channel from skeletal muscle sarcoplasmic reticulum. J. Biol. Chem..

[38] Scherer N.M., Deamer D.W. (1986). Oxidative stress impairs the function of sarcoplasmic reticulum by oxidation of sulfhydryl groups in the Ca^2+^-atpase. Arch. Biochem. Biophys..

[39] Matsuo M. (1993). Oxygen dependency of life-span in the nematode. Comp. Biochem. Physiol. Comp. Physiol..

[40] Zhu X., Heunks L.M., Ennen L., Machiels H.A., Van Der Heijden H.F., Dekhuijzen P.N. (2006). Nitric oxide modulates neuromuscular transmission during hypoxia in rat diaphragm. Muscle Nerve.

[41] King-Vanvlack C.E., Curtis S.E., Mewburn J.D., Cain S.M., Chapler C.K. (1995). Role of endothelial factors in active hyperemic responses in contracting canine muscle. J. Appl. Physiol. (1985).

[42] Viner R.I., Ferrington D.A., Williams T.D., Bigelow D.J., Schoneich C. (1999). Protein modification during biological aging: Selective tyrosine nitration of the SERCA2a isoform of the sarcoplasmic reticulum Ca^2+^-atpase in skeletal muscle. Biochem. J..

[43] Meszaros L.G., Minarovic I., Zahradnikova A. (1996). Inhibition of the skeletal muscle ryanodine receptor calcium release channel by nitric oxide. FEBS Lett..

[44] Radak Z., Pucsok J., Mecseki S., Csont T., Ferdinandy P. (1999). Muscle soreness-induced reduction in force generation is accompanied by increased nitric oxide content and DNA damage in human skeletal muscle. Free Radic. Biol. Med..

[45] Wolin M.S., Hintze T.H., Shen W., Mohazzab H.K., Xie Y.W. (1997). Involvement of reactive oxygen and nitrogen species in signalling mechanisms that control tissue respiration in muscle. Biochem. Soc. Trans..

[46] Rui T., Kvietys P.R. (2005). NFκB and AP-1 differentially contribute to the induction of mn-sod and enos during the development of oxidant tolerance. FASEB J..

[47] Radak Z., Apor P., Pucsok J., Berkes I., Ogonovszky H., Pavlik G., Nakamoto H., Goto S. (2003). Marathon running alters the DNA base excision repair in human skeletal muscle. Life Sci..

[48] Radak Z., Naito H., Kaneko T., Tahara S., Nakamoto H., Takahashi R., Cardozo-Pelaez F., Goto S. (2002). Exercise training decreases DNA damage and increases DNA repair and resistance against oxidative stress of proteins in aged rat skeletal muscle. Pflugers Arch..

[49] Radak Z., Kumagai S., Nakamoto H., Goto S. (2007). 8-Oxoguanosine and uracil repair of nuclear and mitochondrial DNA in red and white skeletal muscle of exercise-trained old rats. J. Appl. Physiol. (1985).

[50] Radak Z., Atalay M., Jakus J., Boldogh I., Davies K., Goto S. (2009). Exercise improves import of 8-oxoguanine DNA glycosylase into the mitochondrial matrix of skeletal muscle and enhances the relative activity. Free Radic. Biol. Med..

[51] Radak Z., Takahashi R., Kumiyama A., Nakamoto H., Ohno H., Ookawara T., Goto S. (2002). Effect of aging and late onset dietary restriction on antioxidant enzymes and proteasome activities, and protein carbonylation of rat skeletal muscle and tendon. Exp. Gerontol..

[52] Clavel S., Coldefy A.S., Kurkdjian E., Salles J., Margaritis I., Derijard B. (2006). Atrophy-related ubiquitin ligases, atrogin-1 and MuRF1 are up-regulated in aged rat *Tibialis anterior* muscle. Mech. Ageing Dev..

[53] Raue U., Slivka D., Jemiolo B., Hollon C., Trappe S. (2007). Proteolytic gene expression differs at rest and after resistance exercise between young and old women. J. Gerontol. A Biol. Sci. Med. Sci..

[54] Husom A.D., Peters E.A., Kolling E.A., Fugere N.A., Thompson L.V., Ferrington D.A. (2004). Altered proteasome function and subunit composition in aged muscle. Arch. Biochem. Biophys..

[55] Radak Z., Kaneko T., Tahara S., Nakamoto H., Ohno H., Sasvari M., Nyakas C., Goto S. (1999). The effect of exercise training on oxidative damage of lipids, proteins, and DNA in rat skeletal muscle: Evidence for beneficial outcomes. Free Radic. Biol. Med..

[56] Reid M.B. (2005). Response of the ubiquitin-proteasome pathway to changes in muscle activity. Am. J. Physiol. Regul. Integr. Comp. Physiol..

[57] Sultan K.R., Dittrich B.T., Leisner E., Paul N., Pette D. (2001). Fiber type-specific expression of major proteolytic systems in fast- to slow-transforming rabbit muscle. Am. J. Physiol. Cell Physiol..

[58] Kozlovsky N., Rudich A., Potashnik R., Bashan N. (1997). Reactive oxygen species activate glucose transport in l6 myotubes. Free Radic. Biol. Med..

[59] Sandstrom M.E., Zhang S.J., Bruton J., Silva J.P., Reid M.B., Westerblad H., Katz A. (2006). Role of reactive oxygen species in contraction-mediated glucose transport in mouse skeletal muscle. J. Physiol..

[60] Higaki Y., Mikami T., Fujii N., Hirshman M.F., Koyama K., Seino T., Tanaka K., Goodyear L.J. (2008). Oxidative stress stimulates skeletal muscle glucose uptake through a phosphatidylinositol 3-kinase-dependent pathway. Am. J. Physiol. Endocrinol. Metab..

[61] Kellogg D.L., McCammon K.M., Hinchee-Rodriguez K.S., Adamo M.L., Roman L.J. (2017). Neuronal nitric oxide synthase mediates insulin- and oxidative stress-induced glucose uptake in skeletal muscle myotubes. Free Radic. Biol. Med..

[62] Ding H., Heng B., He W., Shi L., Lai C., Xiao L., Ren H., Mo S., Su Z. (2016). Chronic reactive oxygen species exposure inhibits glucose uptake and causes insulin resistance in C2C12 myotubes. Biochem. Biophys. Res. Commun..

[63] White A.T., Schenk S. (2012). NAD^+^/NADH and skeletal muscle mitochondrial adaptations to exercise. Am. J. Physiol. Endocrinol. Metab..

[64] Edington D.W., McCafferty W.B. (1973). Mitochondrial size distribution analysis in the soleus muscle of trained and aged rats. Experientia.

[65] Gariani K., Menzies K.J., Ryu D., Wegner C.J., Wang X., Ropelle E.R., Moullan N., Zhang H., Perino A., Lemos V. (2016). Eliciting the mitochondrial unfolded protein response by nicotinamide adenine dinucleotide repletion reverses fatty liver disease in mice. Hepatology.

[66] Hirschey M.D., Shimazu T., Goetzman E., Jing E., Schwer B., Lombard D.B., Grueter C.A., Harris C., Biddinger S., Ilkayeva O.R. (2010). SIRT3 regulates mitochondrial fatty-acid oxidation by reversible enzyme deacetylation. Nature.

[67] Radak Z., Bori Z., Koltai E., Fatouros I.G., Jamurtas A.Z., Douroudos I.I., Terzis G., Nikolaidis M.G., Chatzinikolaou A., Sovatzidis A. (2011). Age-dependent changes in 8-oxoguanine-DNA glycosylase activity are modulated by adaptive responses to physical exercise in human skeletal muscle. Free Radic. Biol. Med..

[68] Vargas-Ortiz K., Perez-Vazquez V., Figueroa A., Diaz F.J., Montano-Ascencio P.G., Macias-Cervantes M.H. (2018). Aerobic training but no resistance training increases SIRT3 in skeletal muscle of sedentary obese male adolescents. Eur. J. Sport Sci..

[69] Palacios O.M., Carmona J.J., Michan S., Chen K.Y., Manabe Y., Ward J.L., Goodyear L.J., Tong Q. (2009). Diet and exercise signals regulate SIRT3 and activate AMPK and PGC-1α in skeletal muscle. Aging (Albany N. Y.).

[70] Lin L., Chen K., Abdel Khalek W., Ward J.L., Yang H., Chabi B., Wrutniak-Cabello C., Tong Q. (2014). Regulation of skeletal muscle oxidative capacity and muscle mass by SIRT3. PLoS ONE.

[71] Fan W., Waizenegger W., Lin C.S., Sorrentino V., He M.X., Wall C.E., Li H., Liddle C., Yu R.T., Atkins A.R. (2017). PPARδ promotes running endurance by preserving glucose. Cell Metab..

[72] Weindruch R. (1995). Interventions based on the possibility that oxidative stress contributes to sarcopenia. J. Gerontol. A Biol. Sci. Med. Sci..

[73] Trifunovic A., Wredenberg A., Falkenberg M., Spelbrink J.N., Rovio A.T., Bruder C.E., Bohlooly Y.M., Gidlof S., Oldfors A., Wibom R. (2004). Premature ageing in mice expressing defective mitochondrial DNA polymerase. Nature.

[74] Hiona A., Sanz A., Kujoth G.C., Pamplona R., Seo A.Y., Hofer T., Someya S., Miyakawa T., Nakayama C., Samhan-Arias A.K. (2010). Mitochondrial DNA mutations induce mitochondrial dysfunction, apoptosis and sarcopenia in skeletal muscle of mitochondrial DNA mutator mice. PLoS ONE.

[75] Martin C., Dubouchaud H., Mosoni L., Chardigny J.M., Oudot A., Fontaine E., Vergely C., Keriel C., Rochette L., Leverve X. (2007). Abnormalities of mitochondrial functioning can partly explain the metabolic disorders encountered in sarcopenic gastrocnemius. Aging Cell.

[76] Jang Y.C., Lustgarten M.S., Liu Y., Muller F.L., Bhattacharya A., Liang H., Salmon A.B., Brooks S.V., Larkin L., Hayworth C.R. (2010). Increased superoxide in vivo accelerates age-associated muscle atrophy through mitochondrial dysfunction and neuromuscular junction degeneration. FASEB J..

[77] Degens H. (2010). The role of systemic inflammation in age-related muscle weakness and wasting. Scand. J. Med. Sci. Sports.

[78] Ji L.L. (2001). Exercise at old age: Does it increase or alleviate oxidative stress?. Ann. N. Y. Acad. Sci..

[79] McArdle A., Jackson M.J. (2017). The role of attenuated redox and heat shock protein responses in the age-related decline in skeletal muscle mass and function. Essays Biochem..

[80] Marzetti E., Calvani R., Cesari M., Buford T.W., Lorenzi M., Behnke B.J., Leeuwenburgh C. (2013). Mitochondrial dysfunction and sarcopenia of aging: From signaling pathways to clinical trials. Int. J. Biochem. Cell Biol..

[81] Rahman M., Halade G.V., El Jamali A., Fernandes G. (2009). Conjugated linoleic acid (CLA) prevents age-associated skeletal muscle loss. Biochem. Biophys. Res. Commun..

[82] Beavers K.M., Beavers D.P., Serra M.C., Bowden R.G., Wilson R.L. (2009). Low relative skeletal muscle mass indicative of sarcopenia is associated with elevations in serum uric acid levels: Findings from nhanes III. J. Nutr. Health Aging.

[83] Lightfoot A.P., McCormick R., Nye G.A., McArdle A. (2014). Mechanisms of skeletal muscle ageing; avenues for therapeutic intervention. Curr. Opin. Pharmacol..

[84] Zhao J., Tian Z., Kadomatsu T., Xie P., Miyata K., Sugizaki T., Endo M., Zhu S., Fan H., Horiguchi H. (2018). Age-dependent increase in angiopoietin-like protein 2 accelerates skeletal muscle loss in mice. J. Biol. Chem..

[85] Ziaaldini M.M., Koltai E., Csende Z., Goto S., Boldogh I., Taylor A.W., Radak Z. (2015). Exercise training increases anabolic and attenuates catabolic and apoptotic processes in aged skeletal muscle of male rats. Exp. Gerontol..

[86] Siu P.M., Alway S.E. (2006). Aging alters the reduction of pro-apoptotic signaling in response to loading-induced hypertrophy. Exp. Gerontol..

[87] Koltai E., Bori Z., Chabert C., Dubouchaud H., Naito H., Machida S., Davies K.J., Murlasits Z., Fry A.C., Boldogh I. (2017). SIRT1 may play a crucial role in overload-induced hypertrophy of skeletal muscle. J. Physiol..

